# Successful diagnosis of intraductal mural nodule in non-dilated main pancreatic duct using novel ultrathin pancreatoscope

**DOI:** 10.1055/a-2717-1508

**Published:** 2025-11-04

**Authors:** Yuta Kawase, Hideyuki Shiomi, Ryota Yoshioka, Ryota Nakano, Takashi Nishimura, Hirayuki Enomoto, Hiroshi Kono

**Affiliations:** 1 26289 Division of Hepatobiliary and Pancreatic Diseases, Department of Gastroenterology, Hyogo Medical University Hospital, Nishinomiya, Japan; 2 26289 Department of Diagnostic Pathology, Hyogo Medical University Hospital, Nishinomiya, Japan

Intraductal papillary mucinous neoplasm (IPMN) is a pancreatic cystic lesion recognized as a precursor for pancreatic adenocarcinoma 1 2 . The presence of a mural nodule is associated with a high malignant potential, and direct endoscopic visualization using disposable pancreatoscopy facilitates accurate diagnosis 3 . However, conventional pancreatoscopes typically have a diameter of 10 Fr, which precludes their insertion into a non-dilated main pancreatic duct (MPD). Herein, we report a case in which successful visualization and diagnosis of an intraductal mural nodule were achieved using a novel ultrathin pancreatoscope for the non-dilated MPD ( Fig. 1 ).

**Fig. 1:**
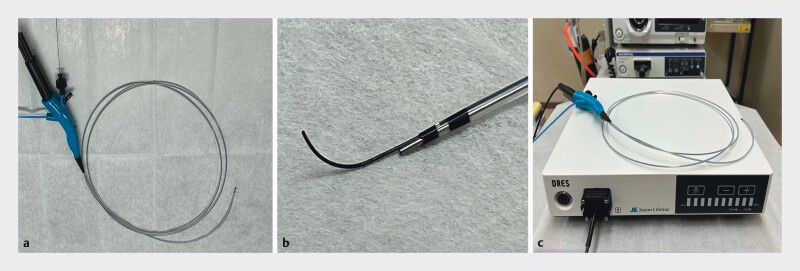
A novel ultrathin pancreatoscope (DRES Slim Scope; Japan Lifeline, Tokyo, Japan). **a** The device features a 4.4-Fr tapered tip with a 1950-mm working length. **b** Independent channel port enabling simultaneous irrigation and 0.025-inch guidewire passage. **c** Compact system setup with a dedicated light source.

A 47-year-old woman was found to have a pancreatic cystic lesion during abdominal ultrasonography. Subsequent magnetic resonance cholangiopancreatography revealed a 19-mm cystic lesion in the pancreatic head with evident communication with the MPD ( Fig. 2 **a** ). Endoscopic ultrasonography demonstrated a mural nodule or mucin plug within the MPD, a finding not clearly apparent on other imaging modalities ( Fig. 2 **b** ). Endoscopic retrograde cholangiopancreatography (ERCP) was then performed for further evaluation. Pancreatography revealed a filling defect in the MPD at the pancreatic head ( Fig. 2 **c** ). A novel ultrathin pancreatoscope (DRES Slim Scope; Japan Lifeline, Tokyo, Japan) was inserted over a guidewire without endoscopic papillary interventions, and direct endoscopic visualization confirmed a villous, protruding lesion within the MPD ( Video 1 , Fig. 3 ). These findings enabled a definitive diagnosis of IPMN with the mural nodule. There were no adverse events, particularly not post-ERCP pancreatitis.

**Fig. 2:**
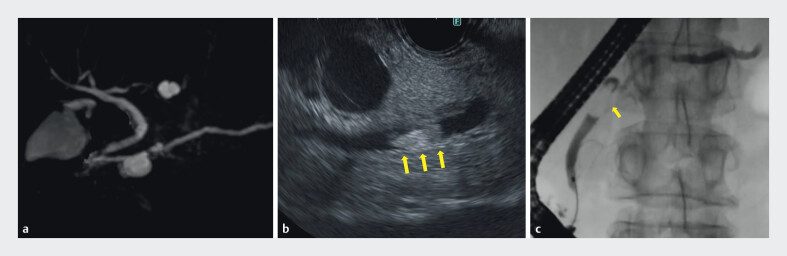
Imaging characteristics. **a** MRCP: a 19-mm cystic lesion in the pancreatic head communicating with the main pancreatic duct (MPD). **b** EUS: the mural nodule within the MPD (yellow arrows). **c** Pancreatography: filling defects in the MPD at pancreatic head (yellow arrow). EUS, endoscopic ultrasonography; MRCP, magnetic resonance cholangiopancreatography.

An ultrathin pancreatoscope (DRES Slim Scope, 4.4-Fr) successfully visualized an intraductal papillary-villous lesion within a non-dilated main pancreatic duct, confirming the nature of a filling defect identified on pancreatography.Video 1

**Fig. 3:**
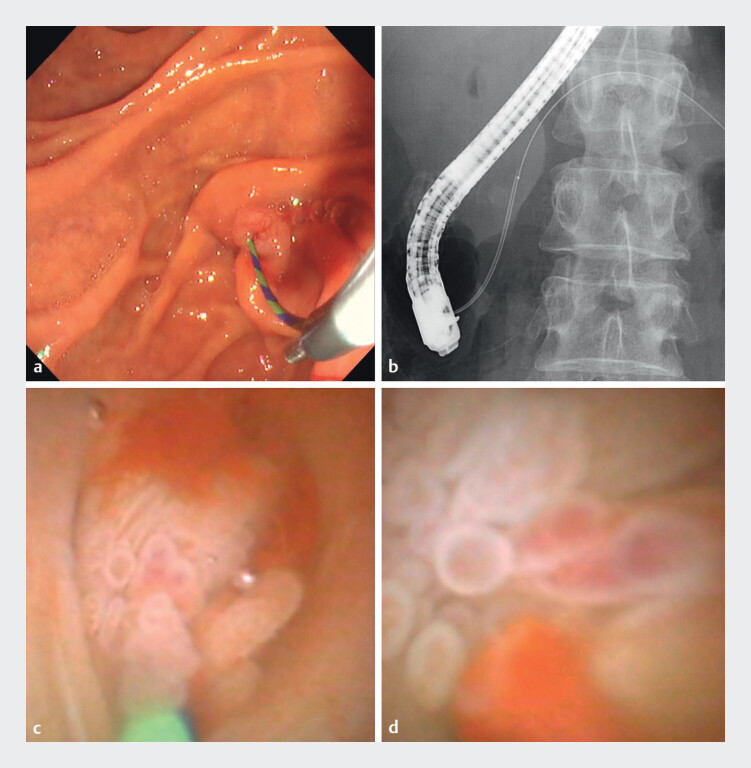
Pancreatoscopic examination. **a** Guidewire-assisted insertion of an ultrathin pancreatoscope. **b** Fluoroscopic visualization of scope advancement into the pancreatic duct. **c** and **d** Direct visualization of an intraductal papillary-villous lesion corresponding to the filling defect.

The ultrathin pancreatoscope used in this case has a diameter comparable to conventional ERCP catheters, allowing insertion into the non-dilated MPD without the need for papillary interventions such as sphincterotomy or balloon dilation. Furthermore, it incorporates an independent channel port, facilitating both irrigation and passage of a 0.025-inch guidewire 4 . This innovative device offers enhanced procedural safety and convenience while lowering the technical barriers to pancreatoscopy. Consequently, it may significantly improve diagnostic capabilities for pancreatic ductal pathologies.

Endoscopy_UCTN_Code_TTT_1AR_2AK

## Notes

Endoscopy E-Videos https://eref.thieme.de/e-videos
*E-Videos* is an open access online section of the journal *Endoscopy* , reporting on interesting cases and new techniques in gastroenterological endoscopy. All papers include a high-quality video and are published with a Creative Commons CC-BY license. Endoscopy E-Videos qualify for HINARI discounts and waivers and eligibility is automatically checked during the submission process. We grant 100% waivers to articles whose corresponding authors are based in Group A countries and 50% waivers to those who are based in Group B countries as classified by Research4Life (see: https://www.research4life.org/access/eligibility/ ). This section has its own submission website at https://mc.manuscriptcentral.com/e-videos .
